# Changes in Metabolite Profiling and Expression Levels of Key Genes Involved in the Terpenoid Biosynthesis Pathway in Garden Sage (*Salvia officinalis*) under the Effect of Hydrazine Hydrate

**DOI:** 10.3390/metabo13070807

**Published:** 2023-06-29

**Authors:** Mohammed Ali, Aisha M. Abdelkawy, Doaa Bahaa Eldin Darwish, Hanan Ali Alatawi, Dikhnah Alshehri, Hadba Al-Amrah, Fathia A. Soudy

**Affiliations:** 1 Maryout Research Station, Genetic Resources Department, Desert Research Center, 1 Mathaf El-Matarya St., El-Matareya, Cairo 11753, Egypt; 2 Botany and Microbiology Department, Faculty of Science, Al-Azhar University (Girls Branch), Cairo 11751, Egypt; aisha.abdelgalil.5921@azhar.edu.eg; 3 Biology Department, Faculty of Science, University of Tabuk, Tabuk 71491, Saudi Arabia; d_darwish@mans.edu.eg (D.B.E.D.); dalshehri@ut.edu.sa (D.A.); 4 Botany Department, Faculty of Science, Mansoura University, Mansoura 35511, Egypt; 5 Department of Biological Sciences, University Collage of Haqel, University of Tabuk, Tabuk 47512, Saudi Arabia; halatwi@ut.edu.sa; 6 Department of Biological Sciences, Faculty of Science, King Abdulaziz University, Jeddah 21589, Saudi Arabia; hggaber@kau.edu.sa; 7 Genetics and Genetic Engineering Department, Faculty of Agriculture, Benha University, Moshtohor 13736, Egypt; soudy@fagr.bu.edu.eg

**Keywords:** *Salvia officinalis*, hydrazine hydrate (HZ), transcriptional control, chemical mutagens, terpene synthase genes

## Abstract

Mutagenesis is a highly efficient tool for establishing genetic variation and is widely used for genetic enhancement in various plants. The key benefit of mutation breeding is the prospect of enhancing one or several characteristics of a variety without altering the genetic background. In this study, we exposed the seeds of *Salvia officinalis* to four concentrations of hydrazine hydrate (HZ), i.e., (0%, 0.1%, 0.2%, and 0.3%) for 6 h. The contents of terpenoid compounds in the *S. officinalis* plantlets driven from the HZ-treated seeds were determined by GC-MS, which resulted in the identification of a total of 340 phytochemical compounds; 163 (87.48%), 145 (84.49%), 65 (97.45%), and 62 (98.32%), from the four concentrations of HZ (0%, 0.1%, 0.2%, and 0.3%), respectively. Furthermore, we used the qRT-PCR system to disclose the “transcriptional control” for twelve TPS genes related to terpenoid and terpene biosynthesis, namely, *SoGPS*, *SoMYRS*, *SoNEOD*, *SoCINS*, *SoSABS*, *SoLINS*, *SoFPPS*, *SoHUMS*, *SoTPS6*, *SoSQUS*, *SoGGPS*, and *SoGA2*. Altogether, results are likely to ensure some positive relationship between the concentrations of the chemical mutagen HZ used for treating the seeds, the type and amount of the produced terpenes, and the expression of their corresponding genes.

## 1. Introduction

Plant improvement has been the cornerstone of the ever-growing human population’s food security for many years. Despite the availability of large germplasm collections, crop development still depends on effective genetic diversity evaluation [1,2,3]. Furthermore, genetic diversity is essential for crop improvement and climate adaptation, especially in plants with low genetic diversity that are more susceptible to stresses [4,5,6,7,8,9]. Genetic variation is greatly influenced by the degree of DNA damage and the cell’s capacity to repair it [10]. According to estimates made by McCulloch and Kunkel (2008) [11], each day during the normal replication mechanism, each cell experiences between 1000 and 1,000,000 molecular damages. Unrepaired DNA damage has the ability to lead to mutations in somatic or germline cells, which can change both the genotype and phenotype of the cell by impairing protein synthesis’s transcription and translation processes [12]. Alkylating agents, such as hydrazine hydrate, ethyl methane sulphonate (EMS), ethyleneimides, alkyl methane sulphonates, sulphur mustards, methyl methane sulfonate, epoxides, and alkyl nitrosoureas, can be utilized as chemical mutagens [13,14]. In this context, mutagenesis is a highly efficient tool for establishing genetic variation, and it has been widely used for genetic enhancement in many different plants, including; cauliflower (*Brassica oleracea*) [15], dianthus (*Dianthus caryophyllus*) [16], chickpea (*Cicerarietinum* L.) [17], barley (*Hordeumvulgare* L.) [18], Arabidopsis (*Arabidopsis thaliana*) [19], Malaysian rice and Korean commercial rice (*Oryza sativa*) [20,21], and sweet corn (*Zea mays*) [22].

*Salvia officinalis*, an important annual medicinal herb of the Lamiaceae family, is extensively cultivated in Europe, the Middle East, Mediterranean areas, Northern Africa, and North Sinai in Egypt. The active pharmaceutical ingredients of salvia species mainly include (1,8-cineole, sabinene, limonene, a-terpineole, ocimene, myrcene, a- and b-pinene, and caryophyllene) [23,24]. As one of the most important popular Egyptian medicinal plants, *S. officinalis* has been used to treat various diseases because of its antioxidant, choleretic, antihypertension, antitumor, antiulcer, anticancer, antimicrobial, anti-thrombosis, antibacterial, antitumorigenic, anti-inflammatory, and anticoagulant properties [25,26]. Terpenoids or isoprenoids are considered one of the biggest secondary metabolites compounds with various structures and sizes [23,24,27,28]. On the other hand, thousands of terpene and terpenoid compounds, such as hemiterpenes, oxygenated monoterpenes, monoterpene hydrocarbons, sesquiterpene hydrocarbons, oxygenated sesquiterpenes, diterpenes, non-iso-prenoid, and triterpene compounds are derived from mevalonate and non-mevalonate pathways [23,24,29,30,31,32]. Due to terpene compounds being involved in the synthesis of various pharmaceutical ingredients, more attention has been paid to them in different salvia species, such as; *Salvia santolinifolia*, *S. isensis*, *S. hydrangea*, *S. epidermidis*, *S. mirzayanii*, *S. fruticosa*, *S. tomentosa*, *S. officinalis*, *S. chloroleuca*, *S. guaranitica*, *S. lavandulifolia*, *S. przewalskii*, *S. japonica*, *S. macrochlamys*, *S. allagospadonopsis*, *S. recognita*, *S. lavandulaefolia*, *S. lanigeraPoir.*, *S. glabrescens*, *S. aureus*, *S. euphratica*, *S. tuxtlensis*, *S. eremophila*, *S. sclaria*, *Salvia staminea*, *Salvia virgata*, *S. nipponica*, and *Salvia verbenaca* [23,31,32,33].

In this study, we inspected the effect of different concentrations of HZ on the expression levels of various terpene biosynthesis genes and determined the biological effect of HZ on terpene and terpenoid production. These results suggested that each concentration of HZ has various effects on the expression level of every terpene synthases gene and terpene production in *S. officinalis* plants. The results of our investigation identifieda method of determining the suitable concentration from chemical mutagenesisand analyzed the biological effects of chemical mutagens, which will facilitate the use of chemical mutagens for the improvement of the salvia plant through mutation breeding.

## 2. Materials and Methods

### 2.1. Plant Material and Growth Conditions

Seeds of *S. officinalis* were pre-soaked in distilled water for 12 h, then soaked in four different concentrations (0%, 0.1%, 0.2%, and 0.3%) of HZ solution for 6 h by placing seeds in 10 mm × 100 mm petri plates (about 60 seeds per plate in a single layer), as described by [34,35,36]. The seeds from each treatment were then washed with distilled water three times to remove the traces of the HZ solution. The *S. officinalis* seeds were then surface sterilized with 75% (*v*/*v*) ethanol for 1:30 min and then in 2.5% (*v*/*v*) sodium hypochlorite solution for 12 min, thoroughly washed three times with sterilized distilled water, and sown in solid Murashige and Skoog (MS) [37] with pH 5.8 medium containing 30 g L^−1^ sucrose and 2.5 g L^−1^ phytagel. The seeds were incubated in the dark for three days then grown at 23 °C under a photoperiod of 8 h dark and 16 h light (110 µmol m^−2^s^−1^) in a controlled growth chamber until the plantlets were grown for 6 weeks. After sowing, the percentage of surviving seedlings (survival rate, SR) was investigated.

### 2.2. RNA Extraction and cDNA Library Preparation

The total RNAs from the three biological plantlets replicates from each *S. officinalis* line (three plantlets from each treatment) were extracted using the plant TRIzol Reagent (Invitrogen, Carlsbad, CA, USA), according to the manufacturer’s protocol. RNA purity was analyzed using a Nano-Photometer^®^ spectrophotometer (IMPLEN, CA, USA), and the quality was examined on 1.4% agarose gels as described previously [23,24,31,38,39]. For quantitative RT-PCR, the first strand of complementary DNA (cDNA) was synthesized from 1 µg total RNA, which was previously treated with DNase I (Takara), using an M-Malva Reverse Transcriptase (RNase H) kit, according to the manufacturer’s protocol. The second strand of cDNA was synthesized in the presence of DNA Polymerase I and Rnase-H, as described previously [23,24,31,38,39].

### 2.3. Metabolite Extraction from S. officinalis Plantlet after Treatment with Different Concentrationsof HZ

For extraction and analysis of terpenoid compounds from the non-treated (control) and treated *S. officinalis* plantlets under different concentrations from hydrazine hydrate, we followed the procedures described by Ali et al., 2017, 2018, 2021, 2022, 2022, 2022 [23,24,31,38,39]. In short, the plantlets from each *S. officinalis* line (three plantlets from each treatment) were collected. Then all collected samples from non-treated (control) and treated *S. officinalis* plantlets were homogenized in liquid nitrogen and the powder was directly soaked in n-hexane in 60 mL bottles. Then, the bottles were incubated with shaking at 36 °C and 210 rpm for 73 h. Afterward, the solvent was purified using a centrifuge at 5050 rpm for 9 min at 5 °C to remove plant debris. The extract was concentrated and transferred to fresh 1.5 mL crimp neck vial amber glass screw-top vials. The terpenoid content in the extract solution was determined by gas chromatography–mass spectrometer (GC-MS: Shimadzu model GCMS-QP2010 Ultra (Tokyo, Japan) system). Three libraries: NIST Library (2014 edition), Volatile Organic Compounds (VOC) Analysis S/W software, and Wiley GC/MS Library (10th Edition), were used to identify the terpenoid constituents by parallel comparison of terpenoid recorded mass spectra with the data that stored in these previous Libraries [23,24,31,38,39]. All of the experiments were performed simultaneously three times under the same conditions for each isolation technique, with a total GC running time of80 min.

### 2.4. Quantitative Real-Time PCR (qRT-PCR) Analysis

Quantitative real-time PCR was performed using an IQTM5 Multicolor Real-Time PCR Detection System (Bio-Rad, Agitech, New Cairo Cairo, Egypt) as described previously by Ali et al., 2017 [23], with SYBR Green I Mix-Master (Roche Diagnostics Ltd., Lewes, UK) following the manufacturer’s instructions, with a total reaction volume of 20 µL, andgene-specific primers for *SoActin* as, a reference gene, and the other twelve genes involved in the biosynthesis of terpenes: *SoGPS* (geranyldiphosphatesynthase), *SoMYRS* (myrcene/ocimene synthase), *SoNEOD* ((+)-neomenthol dehydrogenase), *SoCINS* (1,8-cineole synthase), *SoSABS* ((+)-sabinene synthase), *SoLINS* ((3S)-linalool synthase), *SoFPPS* (farnesyl pyrophosphate synthase), *SoHUMS* (a-humulene/b-caryophyllene synthase), *SoTPS6* ((−)-germacrene D synthase), *SoSQUS* (squalenemonooxygenase), *SoGGPS* (geranylgeranyl pyrophosphate synthase) and *SoGA2* (gibberellin 2-oxidase) from *S. officinalis*. The primers for these previous genes were designed using the primer designing tools of IDTdna (http://www.idtdna.com/scitools/Applications/RealTimePCR/ (accessed on 25 December 2022); primer sequences are listed in (Supplementary Table S1). The quantitative RT-PCR conditions were set as standard conditions: 97 °C for 3:30 min, 36 cycles of amplification (94 °C for 12 s, 58 °C, or 59 °C, or 60 °C for 30 s, and 72 °C for 22 s), and a final extension at 66 °C for 1 min, then to 65 °C for 5 s, and 95 °C for 5 s). The values are means ± SE of the three replicates normalized using *SoActin* as a reference gene. The relative expression levels were calculated by comparing the cycle thresholds (CTs) of the target genes with that of the reference gene *SoActin* using the 2^−ΔΔCt^ method [23,24,31,38,39]. The sizes of amplification products were 150–161 bp. The quantified data were analyzed using Bio-Rad IQTM 5 Multicolor Real-Time Manager software. Finally, the relative expression levels of *SoGPS*, *SoMYRS*, *SoNEOD*, *SoCINS*, *SoSABS*, *SoLINS*, *SoFPPS*, *SoHUMS*, *SoTPS6*, *SoSQUS*, *SoGGPS*, and *SoGA2* genes were detected.

## 3. Results

### 3.1. Identification of Terpenoid Compounds from S. officinalis Plantlets under Different Concentrationsof HZ by GC-MS

**Figure 1:**
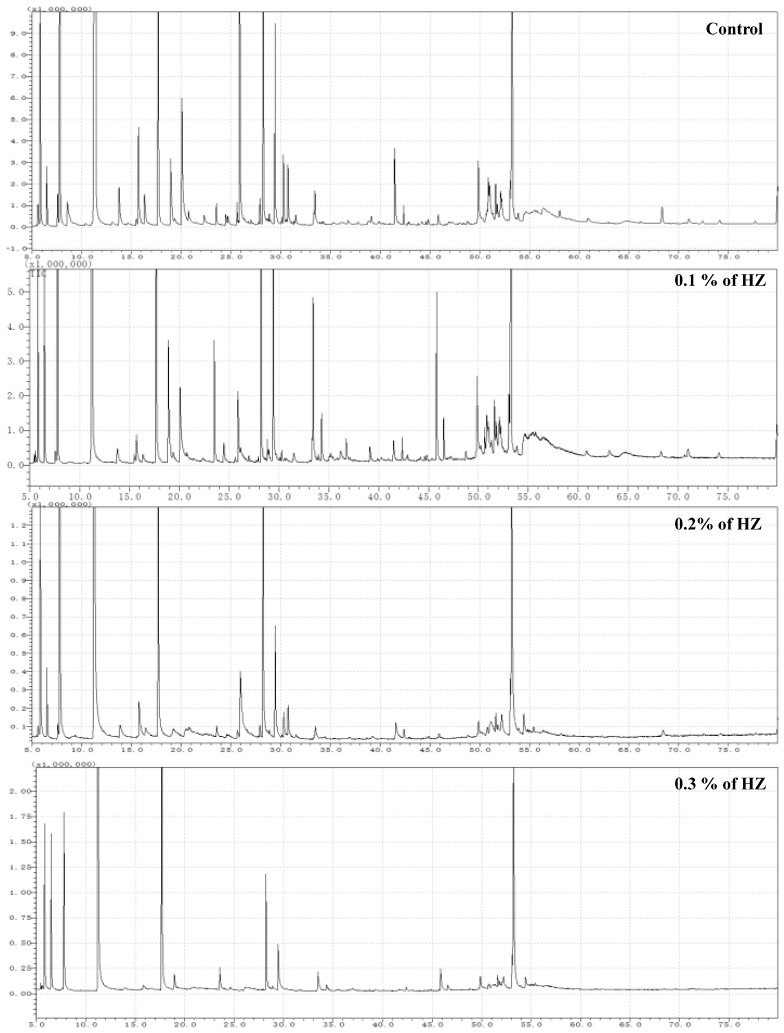
Typical GC-MS mass spectragraphs for terpenoids from *S. officinalis* plantlet under the effect of different concentrations (0%, 0.1%, 0.2%, and 0.3%) of HZ.

To study the effect of HZ on the percentage of survived seedlings, after 6 weeks from plantlets growth, the SR rate was investigated, and we found an inverse relationship between the HZ concentrations and seedlings’ survival rate, which means the SR dramatically decreased with the increase in HZ concentration, and the percentage of SR rate at different concentrations (0%, 0.1%, 0.2%, and 0.3%) of HZ were 51 seedlings (85%), 42 (70%), 33 (55%), and 18 (30%), respectively. Then, the contents of terpenoid compounds in *S. officinalis* plantlets after being treated with different concentrations of HZ were determined by GC-MS, and the results are shown in Figure 1 and Table 1. *S. officinalis* plantlets, after being treated with different concentrations of HZ, produced various levels of mono-, sesquit-, dit-and triterpenes when compared with the control treatment. The numbers of obtained terpenoid and phytochemical compounds from *S. officinalis* plantlets at different concentrations (0%, 0.1%, 0.2%, and 0.3%) of HZ were 163 (87.48%), 145 (84.49%), 65 (97.45%), and 62 (98.32%), respectively. From the GC-MS analysis, we identified 274 phytochemical compounds using n-hexane extracts from the four *S. officinalis* plantlets extracts at different concentrations (0.0%, 0.1%, 0.2%, and 0.3%) of HZ. In *S. officinalis* plantlet extract at 0.0% (control), the monoterpene compounds were shown as the main group (63.3%), followed by the group of sesquiterpene compounds (22.4%) and diterpene compounds (1.78%). At 0.1%, the monoterpene compounds were shown as the main group (52.88%), followed by the group of sesquiterpene compounds (18.13%), then by the group of diterpene compounds (13.41%) and one triterpene compound (0.07%). Monoterpene forms the main group of compounds (78.4%) found in the extract of *S. officinalis* plantlet at 0.2% concentration, followed by the sesquiterpene group (13.27%), diterpenes group (5.78%). Finally, at 0.3% concentration, the monoterpenes compounds were shown as the main group (74.57%), followed by the diterpenes group (14.44%) and sesquiterpene group (9.31%), as shown in Figure 1 and Table 1.

**Figure 2:**
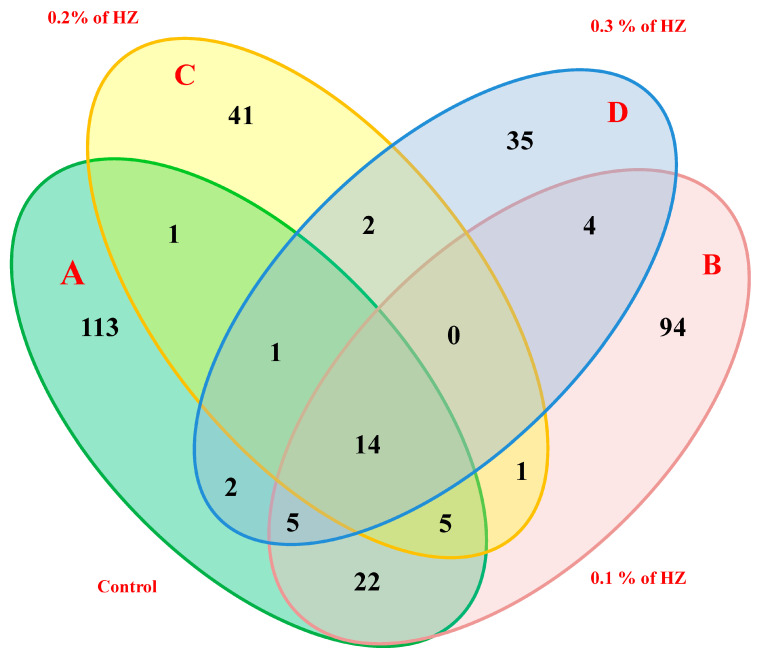
Four-way Venn diagram to show the number of unique and common compounds in the essential oil extracts from *S. officinalis* plantlet under the effect of different concentrations of HZ such as; control (**A**), 0.1% (**B**), 0.2% (**C**), and 0.3% (**D**).

Moreover, the four hexane extracts from the different concentrations (0%, 0.1%, 0.2%, and 0.3%) of HZ have unique, common, and major compounds. For example, the extracts at 0.0% (control) of essential oils (A) had 113 unique compounds, 22 common compounds shared with the extract at 0.1%, 1 common compound shared with the extract at 0.2%, 2 common compounds shared with the extract at 0.3%, and 14 common compounds shared among all 4 HZ concentrations. Furthermore, the extracts at 0.1% concentration (B) contained 94 unique compounds, 1 common compound shared with the extract at 0.2%, and 4 common compounds shared with the extract at 0.3%. In addition, the extracts at 0.2% concentration (C) contained 41 unique, and 2 common compounds shared with the extract at 0.3%. On the other hand, extract at 0.3% (D) contained 35 unique compounds, as reported in (Figure 2).

Regarding the major terpenoid compounds, 1,8-cineole (15.63%) was the major compound in the extracts from *S. officinalis* plantlet at 0.0% concentration, followed by (E)-β-caryophyllene (9.80%), humulene (5.83%), camphor (4.88%), l-2-camphanol e (2.64%), and caryophyllene oxide (1.74%). Whereas the essential oil extract at 0.1% concentration was characterized by 1,8-cineole (35.83%), followed by (E)-β-caryophyllene (9.63%), camphor (8.18%), sugiol (7.76%), α-pinene (3.87%), camphor (2.70%), and humulene (2.46%).

Moreover, 1,8-cineole (47.96%) was the major compound in the extracts from *S. officinalis* plantlet at 0.2% concentration, followed by (E)-β-caryophyllene (8.69%), camphor (6.77%), podocarpa-8,11,13-trien-7-one,12-hydroxy-13-isopropyl (3.89%), α-pinene (3.81%), and 1,4,7,-cycloundecatriene, 1,5,9,9-tetramethyl-,Z,Z,Z (2.50%). In addition, 1,8-cineole (32.32%) was characterized as the major compound in the extracts from *S. officinalis* plantlet at 0.3% concentration, followed by camphor (9.75%), podocarpa-8,11,13-trien-7-one,12-hydroxy-13-isopropyl (6.81%), (E)-β-caryophyllene (6.26%), (+)-camphene (6.02%), β-pinene (3.55%), humulene (4.91%), caryophyllene oxide (1.16%), and sugiol (1.06%) (Table 1). On the other hand, we found fourteen common compounds shared among all four extracts, such as (α-thujene, α-pinene, sabinen, β-pinene, 1,8-cineole, p-menth-8-en-1-ol, stereoisomer, camphor, bornyl acetate, (E)-β-caryophyllene, (+)-germacrene D, caryophyllene oxide, trans-biformene, estra-1,3,5(10)-trien-16-one, 3-((trimethylsilyl)oxy), and ferruginol (Table 1)

When the alignment of the terpenoid composition of the four *S. officinalis* plantlets extracts at different concentrations of HZ, we deduced that some common terpenoid compounds exist at different levels within the four extracts. Therefore, we propose that different concentrations of HZ have a major effect on the kind and level of terpenoid composition in their extract. An important query has been prompted by these data: How does the accumulation of the terpenoid composition in the *S. officinalis* plantlets change depending on the HZ concentration? Before beginning our research, it was difficult to address this question because there was less information at the molecular genetics level regarding the effect of different concentrations of HZ on the terpenoid biosynthetic in *S. officinalis* plantlets.

### 3.2. Overexpressing Terpenoid and Terpene Biosynthesis Genes under the Effect of Different Concentrations of HZ

**Figure 3:**
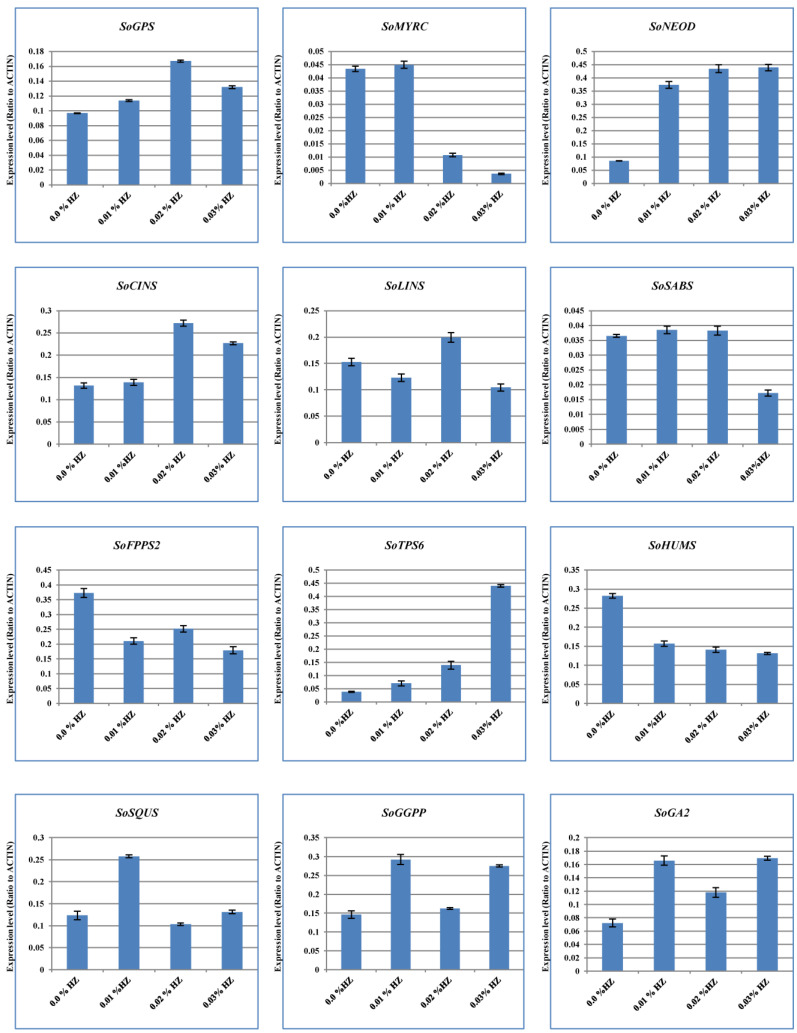
Quantitative RT-PCR validation of expression of terpene synthase genes selected from *S. officinalis* plantlet under the effect of different concentrations of HZ (0%, 0.1%, 0.2%, and 0.3%). Total RNAs were extracted from the previous concentrations, and the expression of *SoGPS*, *SoMYRS*, *SoNEOD*, *SoCINS*, *SoSABS*, *SoLINS*, *SoFPPS*, *SoHUMS*, *SoTPS6*, *SoSQUS*, *SoGGPS*, and *SoGA2* genes were analyzed using quantitative real-time. *SoACTIN* was used as the internal reference. The values are means ± SE of three biological replicates.

To unveil the effects of different concentrations of HZ on various terpenoid and terpene genes expression, we used the qRT-PCR system to measure the level of expression. The results showed that the expression patterns of our candidate genes at different concentrations of HZ (e.g., 0.1%, 0.2%, and 0.3%) were detected, and their expression profiles were compared with the control (0.0% HZ) (Figure 3). For example, the expression levels of *SoFPPS* and *SoHUMS* were the highest under the effect of HZ at 0.0% concentration. Moreover, the highest expression levels for *SoMYRS*, *SoSABS*, *SoSQUS*, and *SoGGPS* were observed at a concentration of 0.1% of HZ. On the other hand, the expression of *SoGPS*, *SoCINS*, and *SoLINS* were the highest in HZ at a concentration of 0.2%. Furthermore, the highest expression levels for *SoNEOD*, *SoTPS6*, and *SoGA2* were detected in a concentration of 0.3% HZ (Figure 3). Therefore, different concentrations of HZ may have an impact on the level of gene expression for genes involved in terpenoid and terpene biosynthesis, according to differences in expression patterns for these genes.

## 4. Discussion

### 4.1. Validation of the Relationship between the Type and Amount of Terpenoid and Gene Expression under Different Concentrations of HZ

We used the qRT-PCR system to reveal the “transcriptional control”, which represents the natural link between the “number of mRNA copies” and “the number of copies of enzyme”, which follow the end-product quantity, in order to ascertain the relationship between the type and amount of terpenoid that was produced under the effect of different concentrations of HZ and gene expression. Therefore, the expression patterns of our twelve candidate genes with various expression levels were detected, and their quantity expression profiles were compared with our GC-MS analysis data. Therefore, according to the gene’s expression levels and the findings of our GC-MS analysis, we found that the *SoGPS* gene showed the highest expression levels at 0.2% concentration, followed by 0.3%, 0.1%, and 0.0% of HZ, and these qRT-PCR results are in line with our GC-MS analysis data, which indicate that the main group of monoterpene compounds was observed at 0.2% concentration, followed by 0.3%, 0.0%, and 0.1% of HZ. Moreover, the highest expression levels for the *SoFPPS2* gene were observed at a concentration of 0.0% of HZ, followed by 0.2%, 0.1%, and 0.3%. In this context, the most abundant sesquiterpene compounds group was detected at a concentration of 0.0% of HZ, followed by 0.1%, 0.2%, and 0.3%.

Additionally, we discovered a favorable link between the gene expression levels of 1, 8-cineole synthase at various concentrations of HZ. For instance, the higher of the 1, 8-cineole synthase gene product and expression level presented at 0.2% concentration, followed by 0.3%, 0.1%, and 0.0%of HZ. In addition, we found an engagement between the (+)-germacrene-D product and germacrene-D-synthase (*SoTPS-6*):the highest product and gene expression were discovered at a concentration of 0.3% of HZ, followed by 0.2%, 0.1%, and 0.0%. Likewise, there is a linkage detected between the (E)-β-caryophyllene, gamma-caryophyllene, humulene, caryophyllene oxide, and 1,2-humulene epoxide as a product and the expression of humulene synthase (*SoHUM*) gene at different concentrations of HZ. Our findings concur with those of other researchers and our earlier research [23,24,31,38,39,40,41,42,43,44,45,46,47], which discovered and discussed a connection between gene expression and the end product, which gives the impression that the production of the terpene compounds under study can be controlled by the gene transcription process.

On the other hand, we discovered that some genes (e.g., *SoMYRS*, *SoSABS*, *SoSQUS*, *SoNEOD*, *SoLINS*, and *SoGA2*) fluctuate in their gene expression level, and also, some terpene compounds (e.g., camphene, geranylisobutyrate, cajeputol, Cis-β-terpineo, thujan-3-one, thujone, l-2-camphanol, ledene, elemene, labda-8(20),14-dien-13-ol, (13R)-, and labda-8(20),14-dien-13-ol, (13R)-) were not detected or were detected in quantities that are not commensurate with the expression levels of their genes under the influence of some concentrations of the HZ. There are numerous explanations for this. First, the expression of some terpene synthase genes may be controlled by the cell’s circadian rhythm. Secondly, the fact that there is a positive correlation between transcript levels and terpene emission which suggests that changes in transcript level are an important determinant of terpene production, even though changes in transcript levels might not directly affect protein levels or enzyme activities due to potential posttranscriptional, post-translational, or enzyme-regulatory mechanisms. Third, the reason why some terpene compounds do not appear may be due to the different rates of protein synthesis, protein modifications, protein degradation, and protein proteolytic turnover. Additionally, by converting some compounds to other compounds through oxidation or glycosylation of monoterpene olefins and sequestration [23,24,48,49]

### 4.2. Assessment of the Effects of Hydrazine Hydrate on the Terpene Genes Expression and Terpenoid Production

Hydrazine hydrate (N_2_H_4_ × H_2_O) is an important inorganic compound, which is mainly used in agrochemicals as a foaming agent [50]. Hydrazine hydrate is considered as one of the alkylating agents (e.g., ethyl methane sulphonate (EMS), ethyleneimides, alkyl methane sulphonates, sulfur mustards, methyl methane sulfonate, epoxides, and alkyl nitrosoureas), which can be used as chemical mutagens [13,14]. Chemical mutagens have been widely used to alter plants’ genetic makeup in various ways, such as: changing the chemistry of the base pairs; nucleotides, and confusing the DNA replication machinery; stripping DNA nucleotides from the essential modifications; in sertion or deletion of some extra base pairs through a round of DNA replication; and two DNA nucleotides cross-linking together, introducing a single base pair (SNPs) [51]. Moreover, the chemical mutagens are usually high in induction and more applicable in the in-vitro compared to physical and radiation approaches [52,53,54,55]. Cells generally attempt to fix these mutations via the cell cycle checkpoints by nucleotide excision repair (NER) and base excision repair (BER) for the eradication of damaged bases and the repair of nucleotides, respectively [52]. Furthermore, in some cases, the disruption of the DNA nucleotide mutation repair mechanism could create mutations in the genetic makeup, which can alter the gene expression and encoding protein and create genetic variability for increased crop productivity through crop improvements, such as that seen in Mung bean (*Phaseolus aureus* Roxb.) [51,56]; for example, EMS, which belongs to the alkylating agents, and is used as a chemical mutagen in plants. EMS has the ability to induce GC → AT transitions in the genomic DNA, which results in mutant proteins that perform alternate roles to those of the normal protein [51]. Additionally, it has been demonstrated that the application of alkylating agents such as EMS and HZ is a practical and efficient way to develop distinctive gene pools in plants [34,35,36,51,56]. Therefore, we can assume that the alkylating compounds are the ones that are used the most commonly use dto cause point mutations, a sort of genetic modification in which only one nucleotide base from an organism’s DNA or RNA sequence is changed, added, or removed [57]. According to the type of point mutation, these changes can have a variety of effects on protein function, composition, and synthesis, ranging from positive effects (synonymous mutations) to negative effects (nonsynonymous mutations) [58,59]. In this context, these mutations can lead to different effects on the differential protein expression levels, such as removing or adding stop codon, which causes the translated protein to be abnormally extended or shortened, change in chemical and physical properties of the amino acids, protein may lose its function and may exhibit a new function or become activated [60,61]. For example, in Yokoyama et al.’s 2022, 2021 study on the stability of mutations in aromatic amino acid (AAAs) compounds from *A. thaliana* seeds, that were mutagenized using EMS [62,63], they isolated a total of 351 suppressor of *tyra2* (sota) mutants that lacked one of two *TyrA* genes that were associated with Tyr biosynthesis—used as a common substrate for the shikimate pathway [62]. This kind of mutant showed an increase in AAAs compared with other amino acids at F1 and F2 of the plant population with dominant or semidominant characteristics, and was accompanied by an increase in net CO_2_ assimilation and flux through the shikimate pathway [62,63,64,65]. These results provide genetic evidence that the induction of point mutations using chemical mutagens has the ability to enhance plant metabolic levels in a dominant fashion.

## 5. Conclusions

The application of chemical mutagens on crops is an easy and effective method for the improvement of various agronomic traits. Seeds from many different plants have been widely used in investigations as the initial materials to produce plant mutations via chemical mutagenesis. There have been fewer studies about the effect of chemical mutagens on medicinal plants because it is difficult to determine the biological and molecular genetic effects of a chemical mutagen on the seeds and plantlets of these plants. This investigation was carried out in order to comprehend the potential impacts of HZ on *S. officinalis* terpenoids and terpene synthesis genes. The goal of the study was to shed light on the effects of four HZ concentrations on the expression levels of the genes involved in terpene synthesis, as well as terpene and terpenoid biosynthesis. The acquired results showed that varied HZ concentrations considerably raised the percentage of most monoterpenes, sesquiterpenes, and diterpenes while increasing the expression levels of twelve terpene genes. The findings of our study open the door to additional research on the use of chemical mutagens to improve a variety of features in medicinal and aromatic plants without changing their genetic makeup. This study might be widely applicable toother salvia species or other genera that principally belong to the Lamiaceae family plants.

## Figures and Tables

**Table 1:** The major chemical composition of the essential oils of *S. officinalis* under the effect of different concentrationsof HZ.

NO.	Compound Name	R.T	Formula	M.W/Da	Terpene Type	Average of% Peak Area				Standard Deviation			
						Control	0.1%	0.2%	0.3%	Control	0.1%	0.2%	0.3%
1	α-thujene	5.577	C_10_H_16_	136.234	Mono	0.12	0.17	0.34	0.78	0.17	0.17	0.16	0.58
2	α-pinene	5.815	C_10_H_16_	136.234	Mono	1.27	3.87	3.81	3.55	2.18	1.36	0.59	1.32
3	camphene	6.477	C_10_H_16_	136.234	Mono	0.21	2.70	1.69	0.82	0.20	2.22	1.88	0.74
4	(+)-camphene	6.49	C_10_H_16_	136.234	Mono	0.76	0.67	1.05	6.02	0.19	0.96	0.25	1.67
5	1,8-cineole	11.298	C_10_H_18_O	154.2493	Mono	15.63	35.83	47.96	32.32	10.93	11.53	5.45	8.86
6	geranylisobutyrate	11.935	C14H24O	208.3398	Sesquit	0.50	0.01	0.00	0.00	0.40	0.01	0.00	0.00
7	cajeputol	11.913	C_10_H_18_O	154.2493	Mono	0.00	0.36	0.00	0.00	0.00	0.09	0.00	0.00
8	P-menth-8-en-1-ol, stereoisomer	13.778	C_10_H_18_O	154.2493	Mono	0.57	0.63	0.37	0.51	0.09	0.40	0.25	0.23
9	Cis-β-terpineo	15.491	C_10_H_18_O	154.2493	Mono	0.07	0.05	0.00	0.00	0.02	0.06	0.00	0.00
10	thujan-3-one	15.706	C_10_H_16_O	152.2334	Mono	0.69	0.81	0.64	0.00	0.62	0.93	0.54	0.00
11	thujone	15.808	C_10_H_16_O	152.2334	Mono	0.39	1.06	0.00	0.72	0.13	0.97	0.00	0.85
12	camphor	17.698	C_10_H_16_O	152.2334	Mono	4.88	8.18	6.77	9.75	4.18	6.65	1.51	2.38
13	l-2-camphanol	18.901	C_10_H_18_O	154.2493	Mono	2.46	1.08	0.00	0.00	4.26	0.61	0.00	0.00
14	(L)-alpha-terpineol	18.93	C_10_H_18_O	154.25	Mono	1.19	0.52	0.00	0.00	1.30	0.70	0.00	0.00
15	alpha terpineol	20.071	C_10_H_18_O	154.25	mono	1.57	0.76	0.00	0.00	1.21	0.52	0.00	0.00
16	2-hydroxy-1,8-cineole	20.735	C_10_H_18_O_2_	170.2487	Mono	0.63	1.46	0.00	0.00	0.56	2.18	0.00	0.00
17	(+)-angelicoidenol	20.832	C_10_H_18_O_2_	170.25	Mono	1.66	0.12	0.00	0.36	2.66	0.19	0.00	0.18
18	2,5-bornanedione	24.456	C_10_H_14_O_2_	166.217	Mono	0.16	0.42	0.10	0.00	0.16	0.42	0.09	0.00
19	cis-2-acetoxy-1,8-cineole	25.609	C_12_H_20_O_3_	212.2854	Mono	0.13	0.18	1.43	0.00	0.18	0.12	2.28	0.00
20	ledene	27.914	C_15_H_24_	204.3511	Sesquit	0.00	0.09	0.16	0.00	0.00	0.16	0.08	0.00
21	(E)-β-caryophyllene	28.208	C_15_H_24_	204.3511	Sesquit	9.80	9.63	8.69	6.26	3.42	5.58	1.43	1.82
22	1H-cycloprop[e]azulene, decahydro-1,1,7-trimethyl-4-methylene-	28.838	C_15_H_24_	204.3511	Sesquit	0.35	0.20	0.00	0.00	0.36	0.11	0.00	0.00
23	gamma-caryophyllene	28.868	C_15_H_24_	204.3511	Sesquit	0.37	0.83	0.00	0.20	0.65	1.44	0.00	0.15
24	humulene	29.416	C_15_H_24_	204.3511	Sesquit	5.83	2.46	0.00	4.91	2.97	2.14	0.00	3.58
25	1,4,7,-cycloundecatriene, 1,5,9,9-tetramethyl-, Z,Z,Z-	29.432	C_15_H_24_	204.3511	Sesquit	0.04	0.10	2.50	0.00	0.08	0.17	0.86	0.00
26	(+)-germacrene D	30.272	C_15_H_24_	204.3511	Sesquit	0.35	0.14	0.34	0.82	0.30	0.01	0.15	0.00
27	elemene	30.723	C_15_H_24_	204.3511	Sesquit	1.19	1.20	0.00	0.00	0.79	1.15	0.00	0.00
28	1-aromadendrene	31.486	C_15_H_24_	204.3511	sesquit	0.13	0.41	0.00	0.00	0.11	0.53	0.00	0.00
29	Cis-muurola-3,5-diene	31.491	C_15_H_24_	204.3511	Sesquit	0.18	0.30	0.00	0.00	0.05	0.39	0.00	0.00
30	spathulenol	33.333	C_15_H_24_	204.3511	Sesquit	0.58	0.37	0.00	0.00	0.67	0.03	0.00	0.00
31	caryophyllene oxide	33.419	C_15_H_24_	204.3511	Sesquit	1.74	1.14	1.38	1.16	1.38	1.05	1.14	0.77
32	1,2-humulene epoxide	34.278	C_15_H_24_O	220.35	Sesquit	0.04	0.54	0.00	0.27	0.00	0.17	0.00	0.17
33	9-hydroxynerol	36.742	C_10_H_18_O_2_	170.25	Mono	0.00	0.35	0.00	0.00	0.00	0.13	0.00	0.00
34	elema-1,3-dien-6.alpha.-ol	36.893	C_15_H_26_O	222.37	Sesquit	1.01	0.00	0.00	0.16	0.99	0.00	0.00	0.06
35	beta.-ylangene	41.488	C_15_H_24_	204.35	Sesquit	0.62	0.28	0.81	0.00	0.42	0.00	1.06	0.00
36	trans-biformene	42.357	C_20_H_32_	272.4681	Diter	0.24	0.22	0.12	0.24	0.10	0.17	0.03	0.12
37	labda-8(20),14-dien-13-ol, (13R)-	45.802	C_20_H_34_O	290.5	Diter	0.00	1.62	0.00	0.00	0.00	1.15	0.00	0.00
38	eudesm-11-en-1-ol	46.516	C_15_H_26_O	222.3663	Sesquit	0.00	0.60	0.00	0.00	0.00	0.04	0.00	0.00
39	viridiflorol	46.574	C_15_H_26_O	222.37	sesquit	0.00	0.00	0.00	0.19	0.00	0.00	0.00	0.03
40	humulane-1,6-dien-3-ol	50.98	C_15_H_26_O	222.3663	Sesquit	0.29	0.60	0.00	0.00	0.22	0.21	0.00	0.00
41	ferruginol	52.105	C_20_H_30_O	286.4516	Diter	0.50	0.53	0.32	0.49	0.34	0.03	0.11	0.29
42	sugiol	53.089	C_20_H_28_O_2_	300.4351	Diter	0.54	7.76	0.00	1.06	0.14	3.24	0.00	0.44
43	podocarpa-8,11,13-trien-7-one, 12-hydroxy-13-isopropyl-	53.27	C_20_H_28_O_2_	300.4351	Diter	0.12	0.00	3.89	6.81	0.21	0.00	3.33	4.86
44	squalene	74.13	C_30_H_50_	410.718	Tri	0.00	0.02	0.00	0.00	0.00	0.04	0.00	0.00

Abbreviations: R.T: retention Time, M.W/Da: molecular weight/Daltons mass.

## Data Availability

All data supporting the findings are available and found in the supplementary data.

## Supplementary Materials

The following are available online at https://www.mdpi.com/article/10.3390/metabo13070807/s1, Table S1: List of *S. officinalis* genes and primer pairs used for qRT-PCR.
