# Supplementary material for: Changes in Metabolite Profiling and Expression Levels of Key Genes Involved in the Terpenoid Biosynthesis Pathway in Garden Sage (Salvia officinalis) under the Effect of Hydrazine Hydrate
Source: Metabolites. 2023 Jun 29;13(7):807. doi: 10.3390/metabo13070807 (PMC10385164; doi:10.3390/metabo13070807)
Supplement: Supplementary file 1 [file metabolites-13-00807-s001.zip › metabolites-2445465-supplementary.pdf]

Supplementary Table S1. List of *S. officinalis* genes and primer pairs used for qRT-PCR.

Gene	Primer name	Primer sequence	PCR product (bp)
SoACTIN	SoACTIN -F	5′- GGCAGTTCTCTCCCTCTAT-3′	157
	SoACTIN-R	5′- GAGGTGGTCGGTGAGAT-3′	
SoGPS	SoGPS-F	5′- CTGGACAAACGGCAGAAG -3′	150
	SoGPS-R	5′- CAATCCCGTGGCGAATATC -3′	
SoMYRC	SoMYRC-F	5′- CACTGCACACGCTATGAA-3′	160
	SoMYRC-R	5′- GTGCTACATGAACGACCATA-3′	
SoNEOD	SoNEOD -F	5′- GTCAATGTCTCCTCCACTTTAG -3′	153
	SoNEOD -R	5′- CTCTTGCAGTTTACCCTCTTT-3′	
SoCINS	SoCINS-F	5′- GGTGTTGCAGGAAGAAGTAG -3′	161
	SoCINS-R	5′- CTGTTGAGTACAGATCCCTTTC -3′	
SoSABS	SoSABS -F	5′- CAACGCCAAAGTTTCGATATCC-3′	150
	SoSABS -R	5′- GCAAGCCTTAAAATCATTCCCG-3′	
SoLINS	SoLINS -F	5′- AGAATTGGTGAAGGCAGAGG-3′	155
	SoLINS -R	5′- GTAGGATGTGGGTCTGATTGG-3′	
SoFPPS2	SoFPPS2-F	5′- CTCTCGGCTGGTGTATTG -3′	159
	SoFPPS2-R	5′- GGATATGGTTCCGGAGAATG -3′	
SoHUMS	SoHUMS-F	5′- GGATGTGTGTAGCCATCTTG-3′	158
	SoHUMS-R	5′- CAAGAGGATGGCTGAGAATG-3′	
SoTPS6	SoTPS6-F	5′- TGAGGATACACTTCAAAGCCC-3′	158
	SoTPS6-R	5′- GTACATCTCAGCCATCCTTATCAT-3′	
SoSQUS	SoSQUS-F	5′- GTATCTCTGTGCTGCTGATG-3′	155
	SoSQUS-R	5′- GCTCTCTCTGTTCTCCTGA-3′	
SoGGPP	SoGGPP-F	5′- GCGGAGATTCTTGATGAGTG-3′	154
	SoGGPP-R	5′- CCGAAATTCCTGAGCTTCTC-3′	
SoGA2	SoGA2-F	5′- GTTCCGTCGTTGTTGCTGAG-3′	156
	SoGA2-R	5′- TAACAACCACCGCCGAAACG-3′	
